# Carbon-Coated Multifunctional Magnetic Nanoparticles for Fluorescent Detection and Removal of β‑Lactam Antibiotics from Water

**DOI:** 10.1021/acsomega.5c11157

**Published:** 2025-12-11

**Authors:** Atailson Oliveira da Silva, Mariana Magalhães Maranhão, Juliani Penha Caland, Guilherme Gomide, Ahmed Subrati, Sergio Enrique Moya, Alex Fabiano Cortez Campos, Marcelo Henrique Sousa

**Affiliations:** † Green Nanotechnology Group, 28127 University of Brasilia, CEP 72220-900 Brasilia, DF, Brazil; ‡ Institute of Physics, University of Brasília, Brasília, DF CEP 70910-900, Brazil; § Complex Fluids Group, Institute of Physics, University of Brasília, CEP 70919-970 Brasilia, DF, Brazil; ∥ Soft Matter Nanotechnology Laboratory, 90216 CIC biomaGUNE, San Sebastian 20009, Guip, Spain; ⊥ Laboratory for Environmental and Applied Nanoscience, FUP, University of Brasília, CEP 73345-010 Planaltina, DF, Brazil; # International Center of Physics, Institute of Physics, University of Brasilia, Brasilia, DF 70910-900, Brazil

## Abstract

The present study addresses the design of an innovative magneto-luminescent nanocomposite by coating cobalt ferrite nanoparticles with an organic layer, enhancing their multifunctional properties. Fourier transform infrared (FTIR) spectroscopy, Raman spectroscopy (Raman), and X-ray photoelectron spectroscopy (XPS) analyses confirm the successful formation of an organic layer rich in functional groups, including hydroxyls, carbonyls, and amines. These groups influenced the surface charge, resulting in positive ζ-potential at acidic pHs and negative ζ-potential at alkaline ones. Nitrogen adsorption measurements and Brunauer–Emmet–Teller (BET) and Barrett–Joyner–Halenda (BJH) analyses revealed a decrease in surface area but an increase in pore size and volume. The nanocomposite particles exhibited a 26% increase in magnetization at room temperature compared to that of cobalt ferrite, attributed to their larger size. Additionally, the organic coating conferred luminescent properties, enabling the detection of β-lactam antibiotics, as evidenced by luminescence enhancement. For amoxicillin, a detection limit of 26 μmol/L (0–500 μmol/L) was determined. The adsorption kinetics experiments conducted with 0.2 g of the nanocomposite per liter of solution with contaminant at room temperature and pH 6.8 indicate that the process follows a pseudo-second-order (PSO) model (*q*_e_ = 149.4 ± 0.4 mg·g^–1^). The thermodynamic tests indicate that the interaction between the nanocomposite and amoxicillin is spontaneous at low temperatures. Finally, the nanocomposite demonstrated the dual capability of detecting and magnetically removing amoxicillin from aqueous solutions, highlighting its potential for environmental remediation applications.

## Graphical abstract

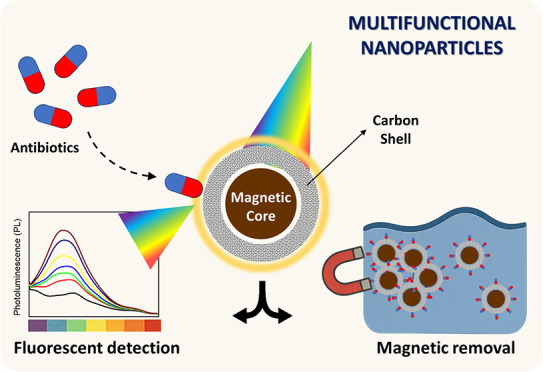

## 1 Introduction

The development of new methods for detecting and removing contaminants from wastewater has become a growing demand in recent years as conventional processes are increasingly costly and ineffective for these purposes, primarily considering the rise in waste volume and the complexity of substances released through anthropogenic processes. In this context, the increasing release of residues, such as pharmaceuticals and their metabolites, into sewage has encouraged scientists, government councils, and researchers to identify and seek more effective water treatment methods to prevent the emergence of new environmental problems. This is especially important, as classical treatment methods cannot ensure the removal or complete degradation of these substances and their derivatives.

In parallel, analytical methods for detecting and quantifying water pollutants are expensive and time-consuming, making it difficult to implement containment barriers and conduct systematic studies on the degradation cycle of substances like antibiotics, anxiolytics, hormones, and analgesics, among others, in wastewater. For instance, amoxicillin, a penicillin-based antibiotic used to treat a wide range of bacterial infections, is excreted in urine at a rate of 80% within 2 h of ingestion.^1^ This, combined with high consumption rates of the drug, has raised concerns due to its direct impact on microorganisms and the potential generation of bacterial resistance.

Currently, high levels of steroid hormones in wastewater have been reported in various regions worldwide,^2^ causing even greater concern. Aquatic organisms are highly vulnerable to the presence of steroid hormones, and reported effects include changes in behavior, development, fertility, and reproductive disorders, which may be associated with alterations in sexual characteristics during early developmental stages.^3^

Nanostructured materials have emerged as valuable tools in developing water treatment solutions, as their chemical and physical characteristics, such as high (functionalized) surface area, and optical and magnetic properties, can be utilized for adsorption, detection, and removal of specific contaminants such as toxic ions, dyes, hormones, and pharmaceuticals.^4^ For this purpose, nanomaterials such as magnetic oxides, quantum dots, carbon nitrides, and carbon-based nanoparticles have been employed either as adsorbents or as detection probes. However, to fully unlock their potential, the development of nanocomposites that combine more than one material with different properties in a single composite is crucial for enhancing both detection and removal capabilities. A key strategy in this process is the surface modification of nanoparticles, which can improve their stability, dispersion in polar or nonpolar solvents, adsorption capacity, and optical properties.^5^

In this context, the main goal of this study is to explore surface modification as a strategy for developing an innovative nanocomposite that integrates magnetic and luminescent properties, with potential applications as both adsorbents and detection sensors. This multifunctional nanomaterial, not yet reported in the literature for these purposes, consists of two distinct components arranged in a core@shell architecture: an inorganic core of cobalt ferrite nanoparticles (CoFe_2_O_4_) with high magnetic responsiveness (MNP), and an organic luminescent coating layer of carbon (C) with surface functional moieties.^6^ The nanocomposite (MNP@C) is fabricated through a two-step process, where the magnetic component is synthesized via coprecipitation, followed by a hydrothermal treatment in the presence of organic precursors to form the carbon shell. The hypothesis underlying this work is that the carbon layer, with fluorescent properties, distinct textural characteristics, and pH-responsive functional chemical groups, enhances the interaction capacity of nanoparticles with inorganic and organic contaminants, thereby improving both the detection and removal efficiency. Additionally, this soft carbon shell acts as a protective barrier, preventing core dissolution and ion leaching into the solution. Meanwhile, the magnetic core enables external field manipulation, facilitating material recovery, an essential feature for pollutant removal in contaminated water. To validate these properties, the nanocomposite underwent comprehensive structural, optical, magnetic, and surface characterization, followed by an evaluation of its ability to quantify pharmaceuticals in solution via fluorescent detection as well as its adsorption efficiency for removing amoxicillin from water through adsorption kinetics experiments.

## 2 Experimental Section

### 2.1 Materials

Ferric chloride hexahydrate (FeCl_3_·6H_2_O) (97%), cobalt nitrate hexahydrate (Co­(NO_3_)_2_·6H_2_O) (98%), ferric nitrate nonahydrate (Fe­(NO_3_)_3_·9H_2_O) (98%), sodium hydroxide (NaOH) (97%), hydrochloric acid 37% (HCl), nitric acid 65% (HNO_3_), acetone (99.5%), d-glucose (96%), ammonium acetate (C_2_H_7_NO_2_) (97%), amoxicillin trihydrate (C_16_H_19_N_3_O_5_S·3H_2_O) (90–100%), ketoprofen (C_16_H_14_O_3_) (98%), tetracycline (C_22_H_24_N_2_O_8_·*x*H_2_O) (98–102%), and cephalexin (C_16_H_17_N_3_O_4_S) (99%) were of analytical grade and purchased from Sigma-Aldrich (São Paulo, Brazil). Ultrapure water (type 1) was used to prepare the solutions.

### 2.2 Sample Synthesis

#### 2.2.1 Magnetic Core (MNP)

Cobalt ferrite (CoFe_2_O_4_) was chosen to fabricate the magnetic core since it possesses enhanced saturation magnetization, which can improve the separation capacity of nanoadsorbents and has already been successfully explored for the removal of contaminants from water.^7,8^ As illustrated in Figure 1, these magnetic nanoparticles were synthesized by coprecipitation followed by a surface treatment, adapted from ref 9. Initially, a 400 mL solution containing a mixture of 1.5 mol/L Fe^3+^ and 0.75 mol/L Co^2+^ (Fe/Co molar ratio = 2) was heated to approximately 80 °C. This solution was then gradually added to a 2 L NaOH solution (4 mol/L), which had been preheated to 100 °C and maintained under constant stirring at 200 rpm. The resulting mixture was stirred continuously for 2 h. After cooling, the precipitate that formed (CoFe_2_O_4_) was magnetically separated and washed three times with ultrapure water to remove residual impurities. Subsequently, the precipitate was acidified by adding 200 mL of HNO_3_ (1 mol/L) and stirring for 10 min, followed by magnetic decantation and discarding of the supernatant.

**Figure 1:**
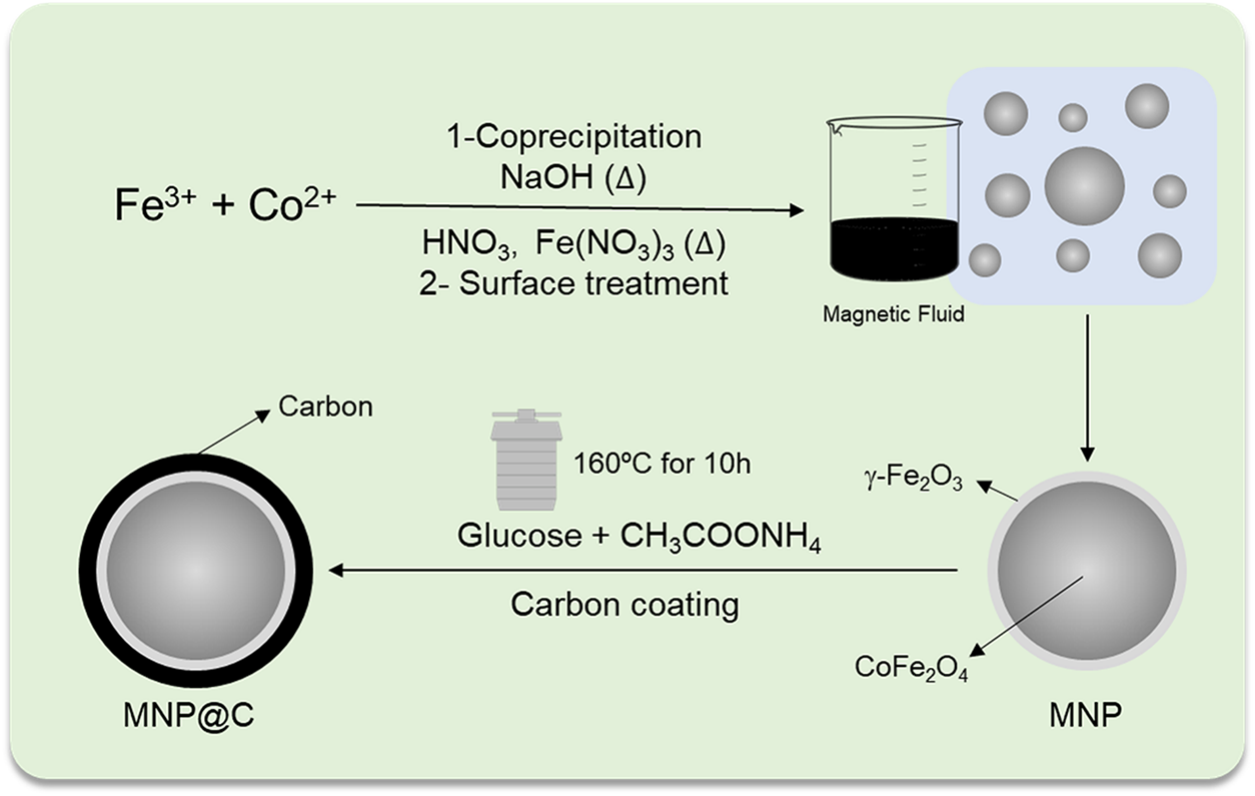
Scheme illustrating the synthesis of carbon-coated magnetic nanoparticles (MNP@C).

In the second step, 150 mL of Fe­(NO_3_)_3_·9H_2_O (1 mol/L) was added to the precipitate, and the system was heated up to boiling while stirring for 20 min. This surface treatment creates a very thin shell of maghemite (γ-Fe_2_O_3_) on the nanoparticle surface, which enhances chemical stability against dissolution and reduces nanoparticle toxicity.^10,11^ Finally, the precipitate was washed three times with acetone and redispersed in water, forming a magnetic fluid with a concentration of 117 mg/mL of the magnetic nanomaterial.

#### 2.2.2 Nanocomposite MNP@C

The synthesis of MNP@C, illustrated in Figure 1, was performed by adapting a synthesis described in the literature.^6^ Briefly, 13 mL of the synthesized magnetic fluid (containing 1.5 g of MNPs) was dispersed in 50 mL of water containing 1.5 g of ammonium acetate under stirring. After 5 min, a 50 mL solution containing 1 g of glucose was added, and the volume was increased to 250 mL, under stirring for a further 10 min. Then, the dispersion was transferred to a Teflon-lined steel autoclave and heated to 160 °C for 10 h. The precipitate formed was separated magnetically, and the supernatant was discarded. Finally, the material was washed three times with distilled water and three more times with acetone. The sample was dried in an oven at 80 °C for 24 h to obtain the powder labeled as MNP@C.

### 2.3 Characterization Methods

The morphology and size of the nanoparticles were evaluated using a JEM-2100F transmission electron microscope (JEOL Ltd., Japan) at an acceleration voltage of 200 kV, employing a carbon-coated copper grid mesh. The crystallographic characteristics were investigated through X-ray diffraction (XRD) powder analyses of the samples using a Rigaku X-ray diffractometer Miniflex 600 with Cu Kα radiation (λ = 1.54 Å). Structural aspects were examined by Fourier transform infrared (FTIR) spectra obtained using a Bruker FTIR VERTEX 70v spectrophotometer and Raman spectra obtained with a confocal microscope model LAbRAM HR Evolution Horiba coupled to a CCD (Charge-Coupled Device), using a green source (532 nm). The optical properties of the nanocomposite were investigated through ultraviolet–visible (UV–vis) absorption of aqueous dispersions recorded on a Hitachi ultraviolet–visible (UV–vis) spectrophotometer model U-3900H, and fluorescence spectra were measured on a Hitachi fluorescence spectrometer model F-7000. The magnetic characterization was carried out as a function of applied field (−7 to 7 T) at both 300 and 5 K using a SQUID (superconducting quantum interference device) magnetometer S700X-R manufactured by Cryogenic. The surface area was evaluated by nitrogen adsorption–desorption isotherms measured with a Micromeritics ASAP-2020 porosimeter, with the samples being degassed in the equipment at 80 °C for 12 h prior. X-ray photoelectron spectra were recorded using a PHI XPS VersaProbe III energy spectrometer equipped with a monochromatic 1486.6 eV Al–Kα radiation source. A focused X-ray source with a 100 μm beam size, 25 W power, and 15 kV e-beam energy was used. Charging neutralization was possible by using a complementary dual-beam charge neutralization method. The C 1s peak at 284.8 eV was used as a reference to calibrate all acquired spectra. The effective surface charge of the composite in aqueous dispersions at varying pH values was evaluated through ζ-potential (ζ) measurements using a Nano ZS Zetasizer coupled with an MPT-2 autotitrator (Malvern). The electrophoretic mobility data were converted into ζ-potential values using Henry’s equation.^12^

### 2.4 Fluorescent Detection

The study of the nanocomposite’s luminescence in the presence of pharmaceuticals was conducted with amoxicillin, ketoprofen, tetracycline, and cephalexin. A constant nanocomposite concentration (20 mg/L) and increasing amounts of drug (0 → 500 μmol/L) were used. The pH of the dispersion was kept constant at 6.8, close to the pH observed in common wastewater, and the spectra were collected under excitation at 320 nm. In all luminescence assays, the analyte was incubated with the nanocomposite for 20 min before the measurements.

### 2.5 Adsorption Kinetics Experiment

To study the adsorption kinetics of amoxicillin on the MNP@C nanocomposite, a mass of 10 mg and an initial amoxicillin concentration of 100 mg/L were placed in a volume of 50 mL. The dispersion was stirred orbitally at 400 rpm, and the pH was maintained constant at 6.8 throughout the study. At 10 min intervals, the system was subjected to an external magnetic field, and after 2 min of application, a 700 mL aliquot of the supernatant was collected for analysis in a UV–vis spectrometer. The amount of amoxicillin adsorbed by the nanocomposite at a time *t* (*q_t_*) expressed in mg/g was calculated using the following expression: $q_{t}=\frac{{(C}_{0}-C_{t})}{m}\times V$ , where *C*_0_ – *C_t_* is the difference between the initial concentration and the concentration of amoxicillin in solution (mg/L) at the time *t*, respectively, and m is the mass of the nanocomposite (mg), and *V* is the volume of the solution.

## 3 Results and Discussion

### 3.1 Sample Characterization

In Figure 2a,b, TEM images of the MNP and MNP@C samples are presented. The size distribution histogram indicates that the magnetic nanoparticles of CoFe_2_O_4_ forming the core have an average size of 7.7 ± 0.2 nm (polydispersity index = 0.42 ± 0.03) and an approximately spherical shape.

**Figure 2:**
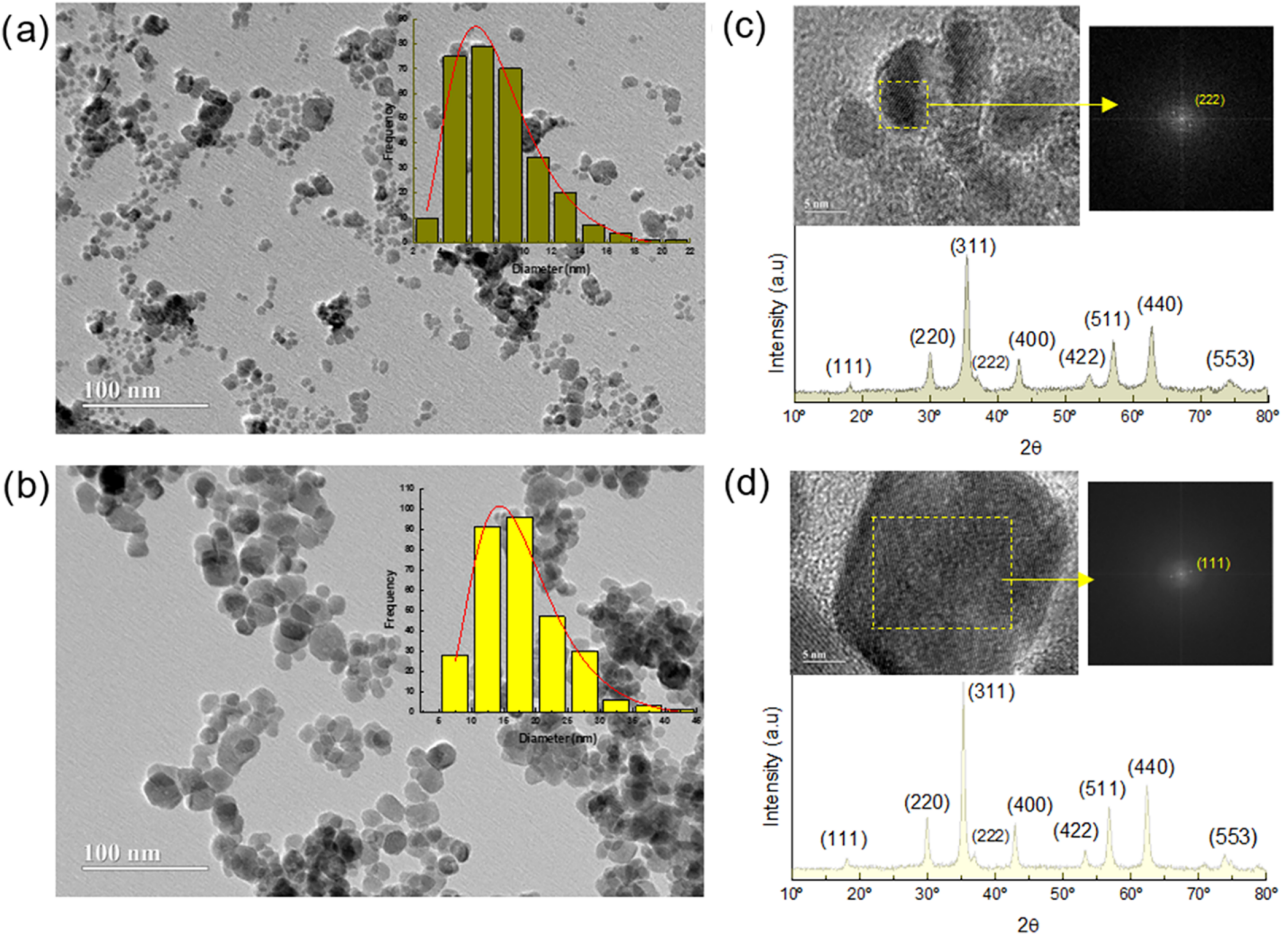
Transmission electron microscopy (TEM) with the size distribution histogram in the top right corner for the MNP sample (a) and MNP@C (b); high-resolution transmission electron microscopy (HRTEM) accompanied by FFT image and X-ray diffractogram with indexed planes for the MNP sample (c) and MNP@C (d).

After hydrothermal treatment with glucose, the particles maintain a nearly spherical morphology; however, the diameter of the nanocomposite increases to 16.9 ± 0.6 nm (polydispersity index *s* = 0.39 ± 0.04). In both distributions, the lognormal fit was applied with *R* ^2^ = 0.98.

From the X-ray diffractograms shown in Figure 2c,d, it is possible to confirm the preservation of crystallinity of the core after hydrothermal treatment, corresponding to the cubic spinel structure of the CoFe_2_O_4_ phase, indexed for both materials using the JCPDS 22–1086.^13^ From high-resolution transmission electron microscopy (HRTEM), the Fast Fourier Transform (FFT) analysis was performed on individual particles, indicating the (222) and (111) planes, respectively, for MNP and MNP@C samples, confirming the spinel structure of ferrite in the core of the nanocomposite. An increase in the crystalline size, from 11 to 16 nm, after hydrothermal treatment was also observed from the narrowing of the (311) XRD peak and calculated by using the Scherrer formalism. This phenomenon has been previously observed and is related to the dissolution of smaller nanoparticles during the hydrothermal treatment with glucose.^6^

In Figure 3a, the Raman spectra for the MNP and MNP@C samples are presented. The peaks between 600 and 800 cm^–1^ originate from the A_1g_ symmetry of the symmetric oxygen–metal stretching in the tetrahedral sites,^14,15^ whereas the peaks at lower wavenumbers, from 200 to 580 cm^–1^, originate from the E_g_ and T_2g_ symmetries corresponding to symmetric and asymmetric oxygen–metal bending in the octahedral sites of the ferrite core. The shift to higher frequency and decrease in intensity in the ferrite characteristic region for the MNP@C sample compared to MNP indicate that the hydrothermal treatment promoted a redistribution of cations in the magnetic core. The Raman spectrum of MNP4@C reveals the formation of an organic carbon layer, evidenced by distinct peaks at 1336 and 1576 cm^–1^. These correspond to the D-band, associated with structural defects and sp^3^-hybridized carbon, and the G-band, indicative of the graphitic sp^2^ carbon network, respectively.^6^ Notably, these bands are absent in the MNP sample. The intensity ratio of the D- to G-band (*I*_D_/*I*_G_ = 0.94) suggests a relatively low defect density within the organic layer.

**Figure 3:**
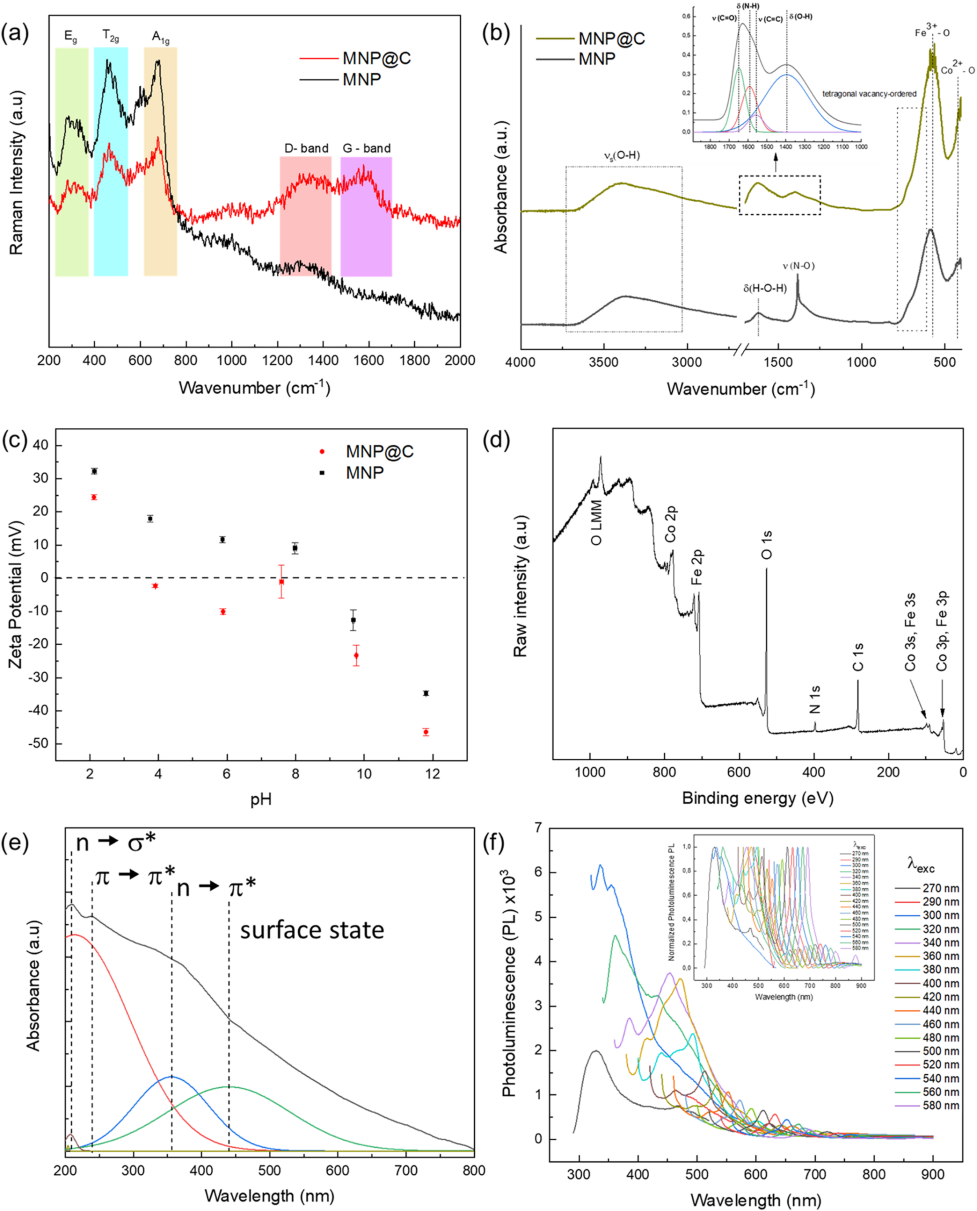
Sample characterization. Raman spectra (a), FTIR spectra (b), and ζ-potential as a function of pH (c) for the MNP@C and MNP samples. XPS survey (d) for the sample MNP@C. UV–vis absorption spectrum (e) and photoluminescence spectrum (f) for the MNP@C sample.

In the FTIR spectra of MNP and MNP@C samples (Figure 3b), a broad absorption band between 3000 and 3700 cm^–1^ originated from the symmetric stretching vibration ν_s_ (O–H) is observed. The strong absorption band at 575 cm^–1^ and a medium vibration at 423 cm^–1^ originate from the stretching vibration ν (Fe^3+^–O) and ν (Co^2+^–O) in the tetrahedral and octahedral sites, respectively, which is typical of cobalt spinel ferrite.^16^ The broadening of the absorption band between 492 and 780 cm^–1^ is due to the occurrence of overlapping peaks.^17^ The region between 1900 and 1000 cm^–1^ highlights the difference between the two samples: for MNP@C, vibration bands ν (C=O) at 1649 cm^–1^, δ (N–H) at 1591 cm^–1^, ν (C=C) at 1560 cm^–1^, and δ (O–H) at 1395 cm^–1^ are identified, which originate from the functional groups on the carbon-based organic layer formed on the magnetic core.

In contrast, for the MNP sample, in the same region, δ (H–O–H) vibrations at 1621 cm^–1^ and ν (N–O) vibrations at 1383 cm^–1^ are identified. The bending vibration of H–O–H in the region between 1600 and 1700 cm^–1^ is observed in hydrated inorganic oxides as cobalt ferrite,^18^ while the stretching vibration of N–O between 1250 and 1500 cm^–1^ originates from residual nitrate ions (–NO_3_^–^) introduced during a stage of the synthesis process.^16^

In Figure S1a (Supporting Information), the N_2_ adsorption–desorption isotherms of the MNP and MNP@C samples were analyzed using Brunauer–Emmet–Teller (BET) and Barrett–Joyner–Halenda (BJH) methods.^19^ The MNP sample exhibits an IUPAC type IV physisorption isotherm profile, typical of materials classified as mesoporous.^20^ This isotherm profile aligns with previously reported data for cobalt ferrite nanoparticles in the literature.^21,22^ After hydrothermal treatment, a change in the physisorption profile of the nanoparticles of MNP@C sample is noticeable, which starts to resemble an IUPAC type II isotherm.^19^

This type of isotherm is observed for nonporous materials or those made up of macropores. The pore distribution obtained by the BJH method is shown in Figure S1b. In line with the differences in hysteresis, a shift toward larger pore diameters is observed in the carbon-coated MNP@C sample compared to the uncapped nanoparticles of the MNP sample.

Table S1 presents the numerical values for the surface area, average diameter, and pore volume of the investigated samples. The surface area of the magnetic core decreases by approximate 30% after carbon coating, yet it remains high compared to uncoated carbon ferrites.^21,23^ This reduction may be explained by the increase in nanoparticle size, as evidenced by the size distribution histogram from TEM images. Interparticle interactions and agglomeration effects play a crucial role in the porosity values measured by the nitrogen powder adsorption method, as interparticle spaces may be considered as pores.^24^ Due to the reduced size and magnetic attraction of nanoparticles, both samples are expected to exhibit large pore size values. However, the significantly higher pore diameter and pore volume values, almost five and three times larger, respectively, suggest that intermolecular interactions are stronger in the carbon-coated sample compared to the uncoated one. This effect is attributed to the presence of functional groups in the organic shell of the coated sample.

The magnetic behavior of the nanomaterials is depicted in Figure S2, and the values extracted from the magnetization curves are collected in Table S2. As observed, the saturation magnetization (*M*_S_) at room temperature for MNP@C and MNP samples was 72.9 and 57.5 Am^2^/kg, respectively, representing a 26% increase in magnetization for the MNP@C sample compared to the uncoated nanoparticles. A similar trend was observed at 5 K, where the coated sample exhibited a 20% higher magnetization than the pure MNPs. The primary factor driving this enhancement in magnetization is the increase in particle size of the carbon-coated nanoparticles, consistent with previous findings.^6^ As a secondary effect, the encapsulation of nanoferrites with organic layers via hydrothermal processes may contribute to the observed improvement in magnetization at room temperature. This improvement could be linked to the chemical reduction of Fe^3+^ ions at the A-sites of the spinel structure,^25^ or the conversion/reduction of γ-Fe_2_O_3_, typically formed during surface treatments synthesis, into magnetically superior Fe_3_O_4_ during hydrothermal treatment.^26^

The room temperature coercive field (*H*_C_) value for MNP@C and MNP samples was 190 and 19 mT, respectively. For the pure nanoparticle, the value is compatible with a significant portion of the sample being superparamagnetic, as previously reported for cobalt ferrite NPs with ∼7 nm size.^27^ As expected for the nanocomposite, due to the increase in nanoparticle size, a greater portion of the sample will be in the blocked state, as observed by the increase in the coercive field at 300 K. At low temperature, this effect gets much clearer, since the difference in coercivity is much smaller, compatible with both samples being almost totally in the blocked stated.^28^ This can be further confirmed by comparing the reduced remanent magnetization (*M*_R_/*M*_S_) for each sample at both temperatures. As shown in Table S2, it decreases to 36% of the value at low temperature for sample MNP@C, while for MNP, it drops to 15%, which confirms that the nanocomposite sample retains a much greater portion of blocked NPs at room temperature. Finally, based on the low temperature measurements, the anisotropy constant of the samples was calculated using the equation $K_{M}=\frac{\mu_{0}M_{S}H_{C}}{0.96}$ . The results indicate an increase of *K*_M_ with hydrothermal treatment, which is compatible with the previously reported trend of increasing anisotropy constant with particle size for cobalt ferrite magnetic nanoparticles up to 20 nm.^29^

The presence of surface charges on nanoparticles dispersed under varying pH conditions is essential for defining their role in the adsorption of molecules and ions. Figure 3c presents the ζ-potential values as a function of pH for both the carbon-coated and uncapped nanoparticles. The mechanism of surface charge generation and the colloidal stability of metal oxide nanoparticles (e.g., CoFe_2_O_4_) in aqueous media is well established in the literature.^30^ Water molecules coordinating with metal sites on the oxide surface (=M–H_2_O, where M represents the metal present on the surface) impart a weak Brønsted acidity. Under strongly acidic pH conditions, the surface sites are protonated (=M–OH_2_^+^), resulting in a positive ζ-potential in this region for the MNP sample. As the pH increases, deprotonation occurs, progressively forming =M–OH species near the point of zero charge. At strongly basic pH, further deprotonation leads to =M–O^–^ species, causing the inversion of the ζ-potential, which becomes negative in this pH region for MNP. The MNP@C nanocomposite exhibits a similar profile of the ζ-potential pH dependence, where the chemical groups from the carbon shell are responsible for the surface charge. In this case, at acidic pH, both carboxylic and nitrogenous groups are protonated −COOH and −NH_2_^+^– (pyrrolic) or −NH^+^= (pyridonic), which results in a positive charge balance. As the pH increases, these groups gradually deprotonate, forming −COO^–^ and −NH– (pyrrolic) or −N= (pyridonic), leading to a predominantly negative charge under strongly alkaline pH^31^ conditions.

Table 1 lists the chemical elements present on the surface of the carbon-coated sample (MNP@C) and their respective percentages, obtained from XPS survey analysis (Figure 3d). As expected, the high percentage of carbon relative to the core elements (Co and Fe) corroborates the successful coating of the magnetic core with an organic layer.

**Table 1:** Surface Element Composition of MNP@C from XPS Analysis

surface element	C	O	N	Fe	Co
(%)	39.37	41.45	3.08	9.92	6.17

Figure S3 presents high-resolution X-ray photoelectron spectra of these elements. After deconvolution of the Fe 3p X-ray photoelectron spectrum (Figure S3.a), the binding energies corresponding to Fe^2+^ were identified in the 3p_1/2_ and 3p_3/2_ regions at 54.96 and 53.76 eV, respectively, and the binding energies for Fe^3+^ in the 3p_1/2_ and 3p_3/2_ regions at 56.75 and 55.55 eV, respectively. The results indicate an Fe^3+^:Fe^2+^ ratio of 2.9:1. Since Fe^2+^ ions were not introduced in the coprecipitation process, it is plausible that under hydrothermal conditions, glucose promoted the reduction of Fe^3+^ ions.^6^ The X-ray photoelectron spectrum of Co 2p (Figure S3.b) suggests the presence of Co^2+^ in the 2p_1/2_ and 2p_3/2_ regions with binding energies at 797.18 and 781.98 eV, respectively, and Co^3+^ in the 2p_1/2_ and 2p_3/2_ regions with binding energies at 795.17 and 779.97 eV, respectively. The peaks at 787.02 and 789.32 eV are associated with the Co 2p_3/2_ satellites, and the peaks at 802.22 and 804.52 eV correspond to the Co 2p_1/2_ satellites.^32^ A strong Fe LMM Auger contribution is identified at 784.54 eV, attributed to the Fe ions in MNP@C. A Co^3+^:Co^2+^ ratio of 1.1:1 was found. The emergence of Co^3+^ ions in cobalt ferrites due to Co^2+^ oxidation by thermal processes has been reported in the literature.^33^ In the deconvoluted O 1s X-ray photoelectron spectrum (Figure S3.c), three binding energies are identified: 529.89, 531.30, and 532.84 eV, corresponding to the M–O (69.7 at. %), C=O (26.6 at. %), and C–O (3.6 at. %) bonds, respectively. For the C 1s X-ray photoelectron spectrum (Figure S3.d), the identified binding energies were 284.74 eV for C=C bonds (56.3 at. %), 285.55 eV for C–C bonds (28.8 at. %), 286.80 eV for C–O/C–N bonds (6.8 at. %), 288.20 eV for C=O/C=N bonds (5.8 at. %), and 289.00 eV for −COO bonds (2.4 at. %). Lastly, in the N 1s X-ray photoelectron spectrum (Figure S3.e), three peaks are observed at 399.10, 400.00, and 400.80 eV, corresponding to pyrrolic N (39.0 at. %), pyridonic N (34.6 at. %), and N–H (26.4 at. %), respectively. These data reinforce the presence of aromatic structures in the organic layer and sites that act as Brønsted bases, as predicted in the ζ-potential analysis.

Figure 3e depicts the UV–vis absorption spectrum of the MNP@C nanocomposite. The absorptions at 208 and 240 nm are attributed to the *n* → σ* and π → π* transitions, respectively, indicating the presence of heterocyclic structures, such as pyrrole, in the organic layer.^34^ The broad absorption band with a peak at 358 nm can be assigned to the *n* → π* transition of the C=O bond. In the low-energy region, a broad absorption band centered at 442 nm is observed, which suggests the presence of different surface states^35^ that give rise to various absorption and emission centers. In Figure 3f, the photoluminescence emission spectra at various excitation wavelengths (λ_exc_ = 270–580 nm) are presented. The data indicate that the MNP@C nanocomposite exhibits luminescence emission with intensity and peak wavelength variations depending on the excitation wavelength. As the excitation shifts to longer wavelengths, a progressive Stokes shift and a decrease in intensity are observed. The highest emission intensity occurs with excitations at 300 and 320 nm, resulting in emissions at 337 and 362 nm, respectively. Emissions of varying intensities appear across the spectrum, and the normalized spectrum reveals broad emission throughout the visible range, spanning from 330 to 829 nm, consistent with UV–vis absorbance data. These results suggest that the carbon organic layer imparts photoluminescent properties to the nanocomposite. The broad emission spectrum is likely arising from diverse surface states or energy traps, a phenomenon originally proposed to explain the luminescent behavior of certain carbon dots.^36^ This interpretation implies that the emission arises not from isolated chemical groups but from the hybridization or organic chemical groups, both within the core (e.g., C=C and C–N) and on the surface (e.g., C=N and C=O) of the carbon phase.

The comprehensive cross-analysis of these characterization results confirms that the nanocomposite, intended for further testing in fluorescent detection and contaminant removal from water, consists of a magnetic core of cobalt ferrite nanoparticles coated with a carbon layer rich in pH-dependent ionizable chemical functional groups.

### 3.2 Fluorometric Determination of Drugs

Building on the luminescent properties of the MNP@C nanocomposite, we investigated its interaction with emerging contaminants, specifically pharmaceuticals. For this purpose, three antibiotics and one nonsteroidal anti-inflammatory drug were selected due to their widespread use: amoxicillin, tetracycline, cephalexin, and ketoprofen, respectively. Their molecular structures are presented in Figure S4.

The photoluminescent response of the nanocomposite as a function of the drug concentration is presented in Figures S5 and 4. The evaluation of photoluminescence in the presence of tetracycline (Figure S5.a) reveals a significant alteration in the emission spectrum with a substantial decrease in intensity even at low concentrations. However, the relationship between *I* – *I*_0_ and the tetracycline concentration does not follow a linear trend (*R* ^2^ = 0.726) (Figure S5b). Consequently, it was not possible to reliably determine the LOD, indicating that the nanocomposite is not suitable as a sensor for tetracycline. Similarly, the nanocomposite exhibited no significant variation in photoluminescence in response to ketoprofen (Figure S5c). The *I* – *I*_0_ vs concentration plot (Figure S5d) shows a plateau across the investigated range (*R* ^2^ = 0.003), further confirming the absence of meaningful correlation, as with tetracycline, the LOD could not be determined, clearly demonstrating that the nanocomposite lacks selectivity and sensitivity for ketoprofen detection.

**Figure 4:**
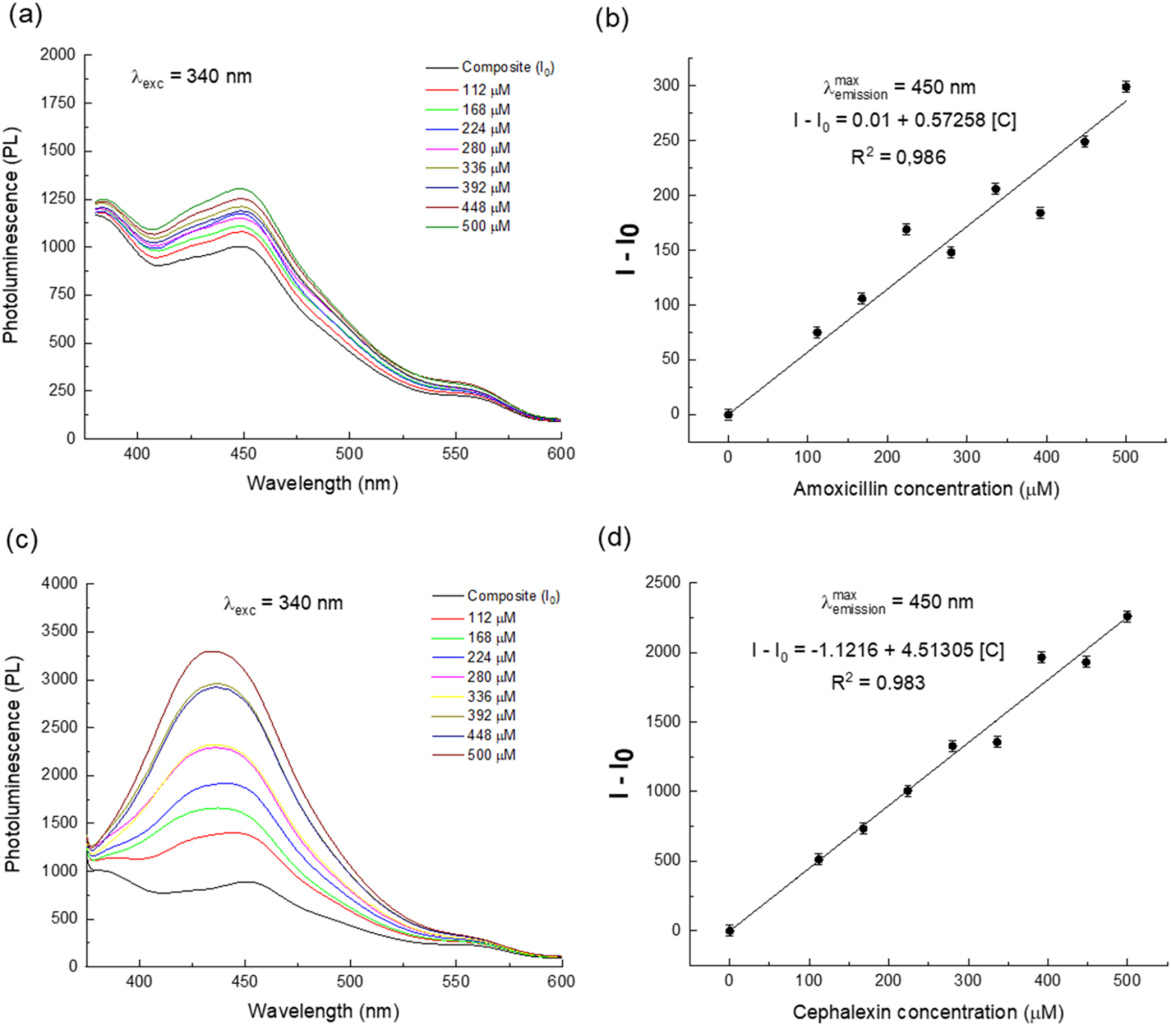
Emission spectrum of MNP@C (λ_exc_ = 340 nm) at different concentrations and plot of the *I* – *I*_0_ as a function of the concentration, respectively, of amoxicillin (a, b) and cephalexin (c, d).

For amoxicillin, an increase in photoluminescence intensity is observed as concentration rises compared to the pure nanocomposite (Figure 4a). The relationship between the increase in luminescence (*I* – *I*_0_) and amoxicillin concentration C shows a notable linearity within the investigated range (*R* ^2^ = 0.986) (Figure 4b), with a limit of detection (LOD) of 26 μmol/L. Similarly, the photoluminescence response of the nanocomposite to increasing cephalexin concentrations (Figure 4c) shows a progressive enhancement in luminescence, with an even greater intensity than that observed for amoxicillin. The plot of photoluminescence increase (*I* – *I*_0_) as a function of cephalexin concentration (Figure 4d) follows a linear trend, with a good correlation (*R* ^2^ = 0.983), and an LOD of 26.6 μmol/L. Both amoxicillin and cephalexin are β-lactam antibiotics, sharing a common β-lactam ring structure composed of three carbon atoms and one nitrogen atom. These preliminary results indicate that the nanocomposite is sensitive to this class of drugs in aqueous media.

Table S3 summarizes previous studies on the nanocomposite-based detection of amoxicillin. Notably, none utilized fluorescence as a detection method, despite its high sensitivity and cost-effectiveness compared to alternative methods. While the MNP@C nanocomposite has not yet achieved detection limits as low as some reported materials, its potential lies in the tunability of its surface properties through controlled chemical modifications during the coating process.

As previously demonstrated, the correlation between amoxicillin concentration and the luminescent intensity increase (*I* – *I*_0_) follows an approximately linear behavior. The enhancement or activation of the luminescence emission is a well-established tool for studying molecular binding to proteins. The equation developed by Bhattacharya et al.^37^ has been used to determine the binding constant for this process: $\frac{1}{\Delta I}=\frac{1}{\Delta I_{\max}}+\frac{1}{K\left[C\right]}\Delta I_{\max}$ , where Δ*I* = *I*_*x*_ – *I*_0_ and Δ*I*_max_ = *I*_∞_ – *I*_0_, with *I*_0_, *I*_*x*_, and *I*_∞_ representing the fluorescence intensity of the nanocomposite in the absence of the analyte, in the presence of the analyte, and at interaction saturation, respectively. The analyte concentration is denoted as [C], while *K* corresponds to the binding affinity constant, whose value is determined from the plot of 1/(*I* – *I*_0_) versus 1/[C]. To elucidate the interaction mechanism between amoxicillin and the nanocomposite, a study was conducted on the variation of luminescence (Δ*I*) as a function of concentration [C] at three different temperatures: 298, 308, and 313 K. Amoxicillin concentrations of 0, 112, 224, 336, and 448 μmol/L were used. Prior to measurements, a thermal bath was used for temperature control, and samples were maintained at the target temperature for 30 min to ensure thermal equilibrium before photoluminescence analysis.

Figure 5a shows the plots of 1/ Δ*I* versus 1/[C] obtaine*d* at different temperatures, adjusted by a linear fitting. The obtained *K* values (in L/μmol) were converted into the dimensionless thermodynamic equilibrium constant *K*_C_ using the equation *K*_C_ = 55.5 · 1 × 10^6^ · K.^38^ The corresponding *K*_C_ and *R* ^2^ values are presented in Table 2. The results indicate that as the temperature increases, the *K*_C_ value decreases, suggesting that higher temperatures weaken the interaction between the nanocomposite and amoxicillin. To further understand the thermodynamic nature of this interaction, the enthalpy change (Δ*H*) and entropy (Δ*S*) values were calculated using the Van’t Hoff equation $\ln K_{C}=-\frac{\Delta H}{\mathrm{RT}}+\frac{\Delta S}{R}$ . A linear fit of ln­(*K*_C_) vs 1/*T* was performed to determine the slope and intercept. From these values, the Gibbs free energy (Δ*S*) was then calculated with the equation Δ*G* = Δ*H* – *T*Δ*S*, where *T* is the thermodynamic temperature and *R* is the gas constant (8.314 J·mol^–1^·K^–1^). The values obtained for Δ*H*, Δ*S*, and Δ*G* of the interaction between the nanocomposite and amoxicillin are given in Table 2.

**Figure 5:**
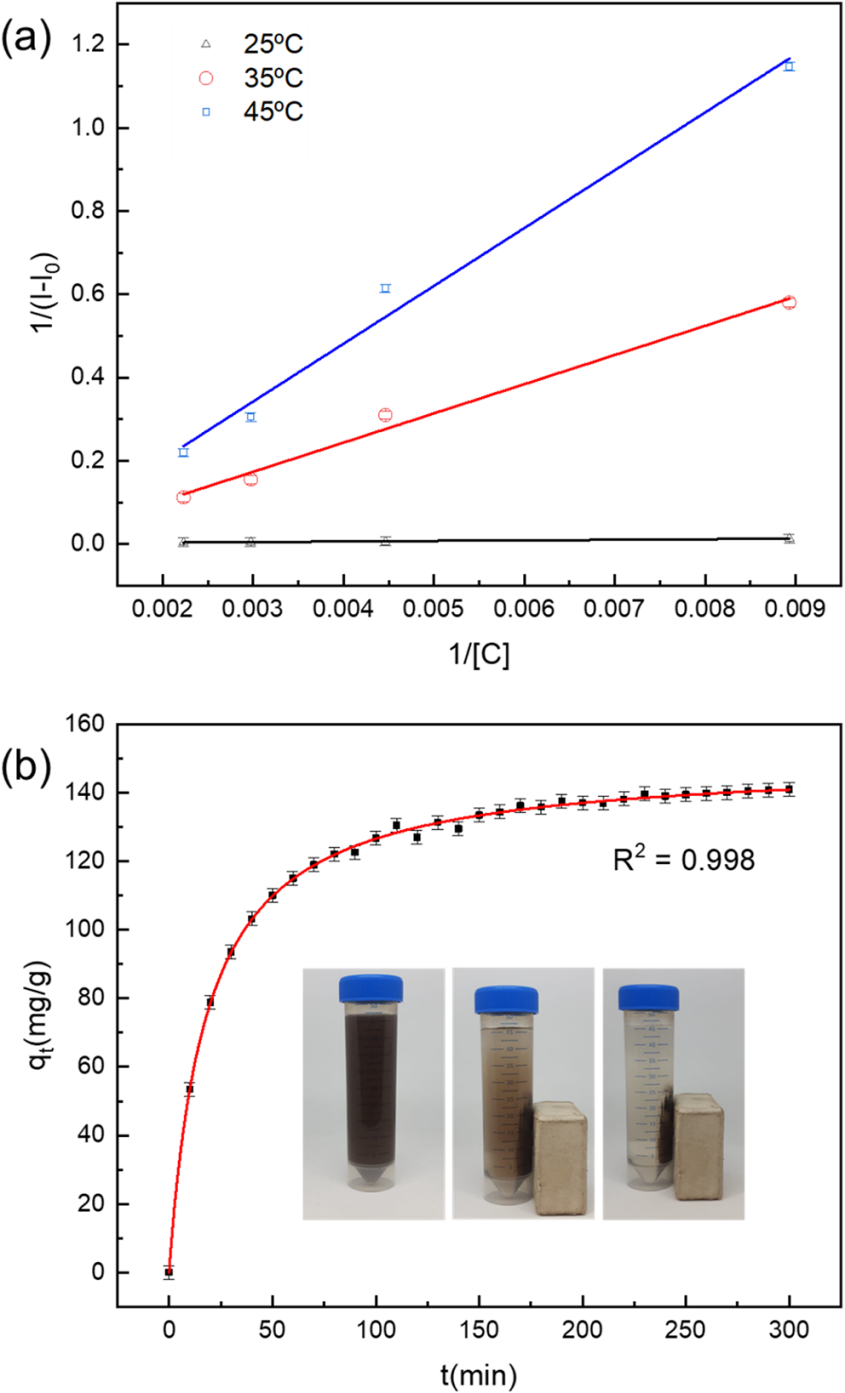
Linear fit of the plots of 1/ Δ*I* versus 1/[C] at different temperatures to determine the value of the binding affinity constant (a). Adsorption kinetics data were fitted with the pseudo-second-order (PSO) model (b). The inset photograph shows the removal of the magnetic nanocomposite under the influence of an external magnetic field in the presence of amoxicillin.

**Table 2:** Binding Equilibrium Constant Values (*K*_C_) and Thermodynamic Parameters Obtained for the Interaction of the MNP@C Nanocomposite and Amoxicillin

*T* (K)	*R* ^2^	*K*_C_	Δ*G* (kJ·mol^–1^)	Δ*H* (kJ·mol^–1^)	Δ*S* (J·K^–1^·mol^–1^)
298	0.982	1.8 × 10^4^	–24.3	–13.5	36.5
308	0.988	1.5 × 10^4^	–24.7		
313	0.987	1.2 × 10^4^	–25.1		

The negative enthalpy change (Δ*H* < 0) indicates that the interaction is exothermic in nature. This is accompanied by an increase in entropy (Δ*S* > 0), indicating greater disorder upon binding. This likely reflects the release of ordered water molecules from the hydration shells of the nanocomposite surface and/or amoxicillin during adsorption, leading to greater freedom of solvent molecules. Furthermore, the negative Gibbs free energy (Δ*G* < 0) confirms that the association process between the nanocomposite and amoxicillin is spontaneous under the studied conditions. This thermodynamic profile indicates that the binding process is driven by both favorable enthalpy (energy decrease) and favorable entropy (increased disorder from water release).

The relationship between the thermodynamic parameters provides insight into the nature of probe-analyte interactions: when Δ*H* < 0, Δ*S* > 0, and Δ*G* < 0, the interaction is primarily electrostatic; when Δ*H* > 0, Δ*S* > 0, and Δ*G* < 0, it is predominantly hydrophobic; and when Δ*H* < 0, Δ*S* < 0, and Δ*G* < 0, van der Waals forces and hydrogen bonding govern the interaction.^39,40^ Given the chemical composition of the nanocomposite surface, which includes hydroxyl, carboxyl, and amine groups capable of acquiring charge depending on the pH of the medium, and considering the thermodynamic data obtained, it is expected that electrostatic interactions play a significant role in the interaction between the nanocomposite and the amoxicillin molecules. However, the potential contribution of hydrogen bonding to the binding mechanism cannot be ruled out.

### 3.3 Adsorption Kinetics

The adsorption kinetics of amoxicillin is depicted in Figure 5b, showing a rapid adsorption rate within the first 30 min, followed by a gradual decrease until equilibrium is reached at approximately 230 min. The inset photograph highlights the efficient magnetically assisted chemical separation of the contaminant-loaded nanocomposites. The experimental data align very well with the pseudo-second-order (PSO) model (*R* ^2^ = 0.998) expressed in the equation $q_{t}=\frac{k_{2}q_{e}^{2}t}{1+k_{2}q_{e}t}$ , where *q*_t_ and *q*_e_ are the amount of amoxicillin adsorbed (mg/g) at time *t* and at the equilibrium, respectively, and *k*_2_ represents the rate constant of the PSO model. From the fitting, *q*_e_ = 149.4 ± 0.4 mg·g^–1^ and *k*_2_ = 3.7 × 10^–4^ ± 6.8 × 10^–6^ g·mg^–1^·min. The approximation equilibrium factor (*R*_w_), which characterizes the kinetic curve in the PSO model, was determined according to Wu et al.^41^ The obtained value of *R*_w_ = 0.073 falls in the range 0.01 < *R*_w_ < 0.1, indicating a highly curved adsorption profile very close to equilibrium. Additionally, the adsorption kinetic data were analyzed using the Elovich and Bangham models (Figure S6). However, both models yielded lower correction coefficients compared to the PSO model.

The electrical charge of amoxicillin varies with the pH of the medium, due to the presence of carboxylic (p*K*_a1_ = 2.68), amine (p*K*_a2_ = 7.49), and hydroxyl (p*K*_a1_ = 9.64) functional groups. At the pH used for the adsorption kinetics study (pH = 6.8), amoxicillin exists in its zwitterionic form, while the nanocomposite surface presents a ζ-potential ranging between 0 and −10 mV. This suggests that mechanisms beyond electrostatic interactions contribute to the adsorption process. For instance, π–π interactions have been reported between amoxicillin and magnetic graphene oxide over a wide pH range.^42^ Additionally, multiple mechanisms, such as electron donor–acceptor interactions and hydrogen bonding, have been observed in the interaction of amoxicillin onto activated carbon.^43^

This study provides the first demonstration of a novel magneto-luminescent nanocomposite with promising multifunctional properties. Its tailored organic surface layer enables both fluorescence-based detection and magnetic recovery, showcasing its potential as a dual-mode platform for environmental analysis. Although preliminary, these findings open new perspectives for multifunctional nanomaterials. Future efforts will aim at optimizing the coating to improve sensitivity, evaluating recyclability, comparing analytical performance with standard techniques, and exploring applicability to other emerging contaminants.

## 4 Conclusions

In this study, we developed a magneto-luminescent nanocomposite by functionalizing a cobalt ferrite magnetic core with an organic surface layer, enhancing both its magnetic and photoluminescent properties. The sensitivity of the nanocomposite to β-lactam antibiotics was confirmed through luminescence enhancement, with a strong linear response observed for amoxicillin and cephalexin. Beyond detection, the nanocomposite exhibited a high adsorption capacity for amoxicillin, reinforcing its potential as a dual-function material for both the identification and removal of emerging contaminants. The efficient separation of contaminant-loaded nanocomposites under an external magnetic field further highlights its practical applicability. Optimizing the surface chemistry by fine-tuning the composition and proportion of functional groups could further improve detection sensitivity and removal efficiency. Moreover, investigating recyclability, benchmarking performance against conventional analytical techniques, and testing with other emerging contaminants would be highly valuable to broaden the applicability of this material in environmental remediation.

## Supplementary Material



## Notes

N_2_ adsorption–desorption isotherm and BJH pore size distribution curves and data of nanocomposites; magnetization curves and data of nanocomposites; XPS high-resolution spectra of nanocomposites; molecular structure of analyzed drugs; emission spectra of drugs; and summary of previous studies on nanocomposite-based detection of amoxicillin (PDF)
