# Supplementary material for: Carbon-Coated Multifunctional Magnetic Nanoparticles for Fluorescent Detection and Removal of β‑Lactam Antibiotics from Water
Source: ACS Omega. 2025 Dec 11;10(50):62358–68. doi: 10.1021/acsomega.5c11157 (PMC12750280; doi:10.1021/acsomega.5c11157)
Supplement: Supplementary file 1 [file ao5c11157_si_001.pdf]

Supporting Information

Carbon-Coated Multifunctional Magnetic Nanoparticles for Fluorescent Detection and Removal of β-Lactam Antibiotics from Water

Atailson Oliveira da Silva[a], Mariana Magalhães Maranhão[a], Juliani Penha Caland[b], Guilherme Gomide[c], Ahmed Subrati[d], Sergio Enrique Moya[d], Alex Fabiano Cortez Campos[e,f], Marcelo Henrique Sousa[a]*

[a] *Green Nanotechnology Group, University of Brasilia, CEP 72220-900, Brasilia-DF, Brazil.*

[b] *Institute of Physics, University of Brasília, Brasília-DF, CEP 70910-900, Brazil*

[c] *Complex Fluids Group, Institute of Physics, University of Brasília, CEP 70919-970, Brasilia, DF, Brazil*

[d] *Soft Matter Nanotechnology Laboratory, CIC biomaGUNE, San Sebastian, 20009, Guip, Spain*

[e] *Laboratory for Environmental and Applied Nanoscience, FUP, University of Brasília, CEP 73345-010 Planaltina, DF, Brazil.*

[f] *International Center of Physics, Institute of Physics, University of Brasilia, Brasilia, DF 70910-900, Brazil*

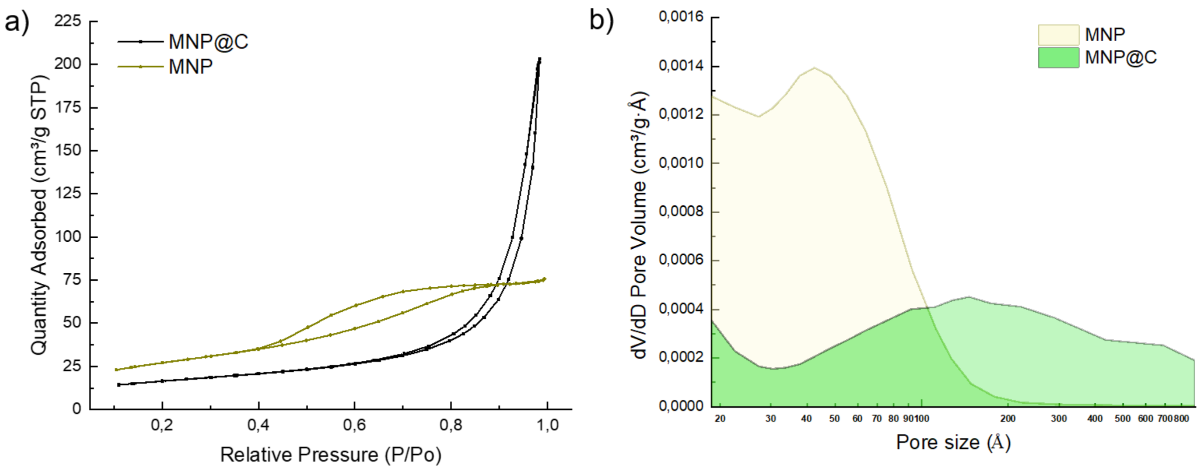

Figure S1. N_2 adsorption-desorption isotherm (a) and BJH pore size distribution (b) for $CoFe_2O_4$ and $CoFe_2O_4$@C samples.

Table S1. Surface area and pore distribution data.

Sample	BET	BJH	
	Surface area [m^2g^{-1}]	Pore diameter [Å]	Pore volume [cm^3 g^{-1}]
MNP	98.5	50.2	0.110
MNP@C	59.4	237.3	0.311

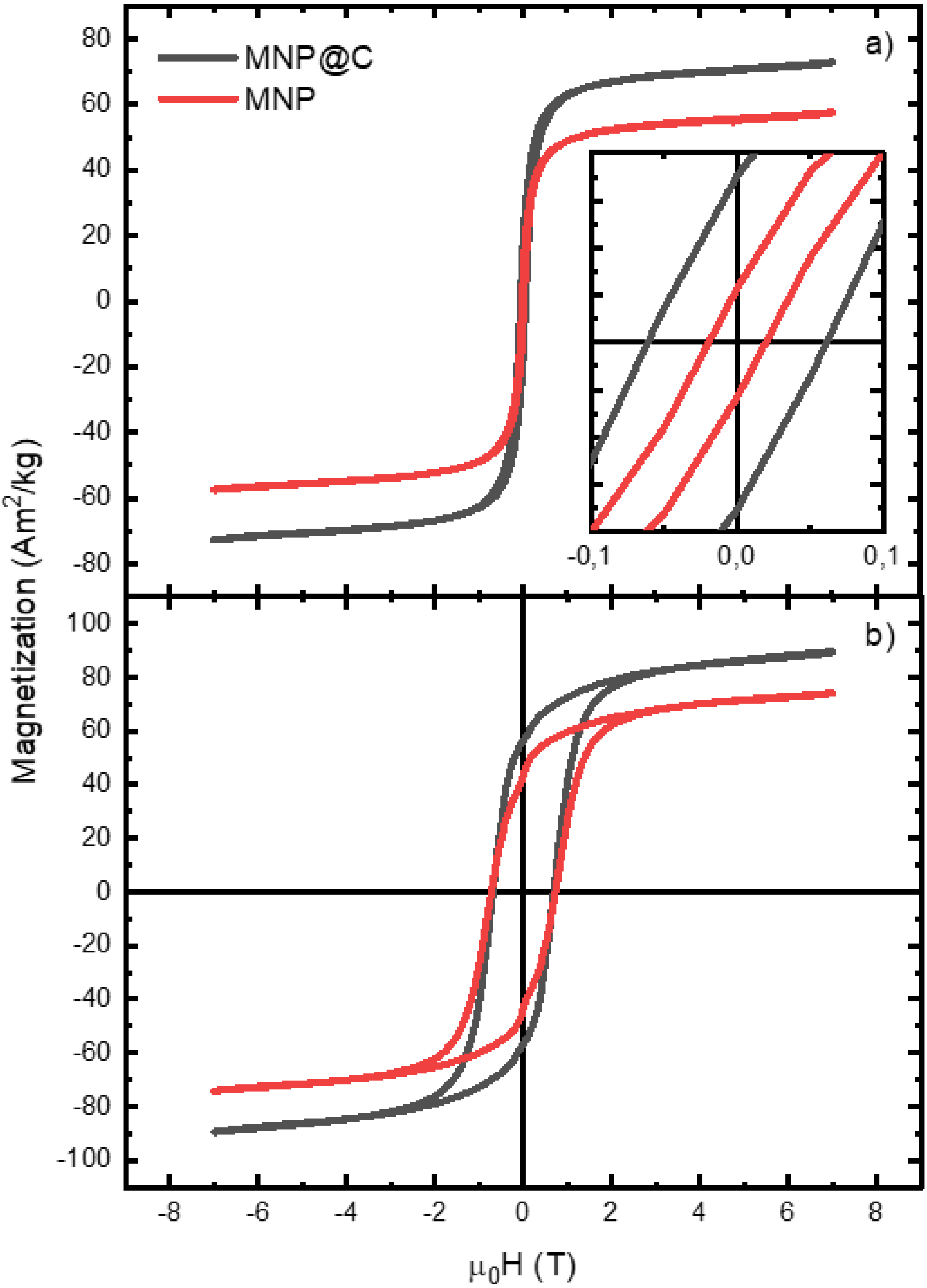

Figure S2. Hysteresis loops recorded between -7 T and 7 T for samples MNP@C and MNP samples at a) 300 K and b) 5K.

Table S2. MNP and MNP@C magnetization data. H_C is the coercive field, M_S the maximum magnetization, obtained at 7 T, M_R the remanent magnetization and K the anisotropy constant.

	**MNP@C**	**MNP**
**H_C 5 K (T)**	0.69	0.73
**H_C 300 K (T)**	0.19	0.019
**M_S 5 K (Am^2/kg)**	89.5	74.1
**M_S 300 K (Am^2/kg)**	72.9	57.5
**M_R 5 K (Am^2/kg)**	58.1	45.3
**M_R 300 K (Am^2/kg)**	17.1	5.5
**M_R/M_S 5 K**	0.65	0.61
**M_R/M_S 300 K**	0.24	0.10
**M_R/M_S (5 K/300 K)**	36%	15%
**K (kJ/m^3)**	322	282

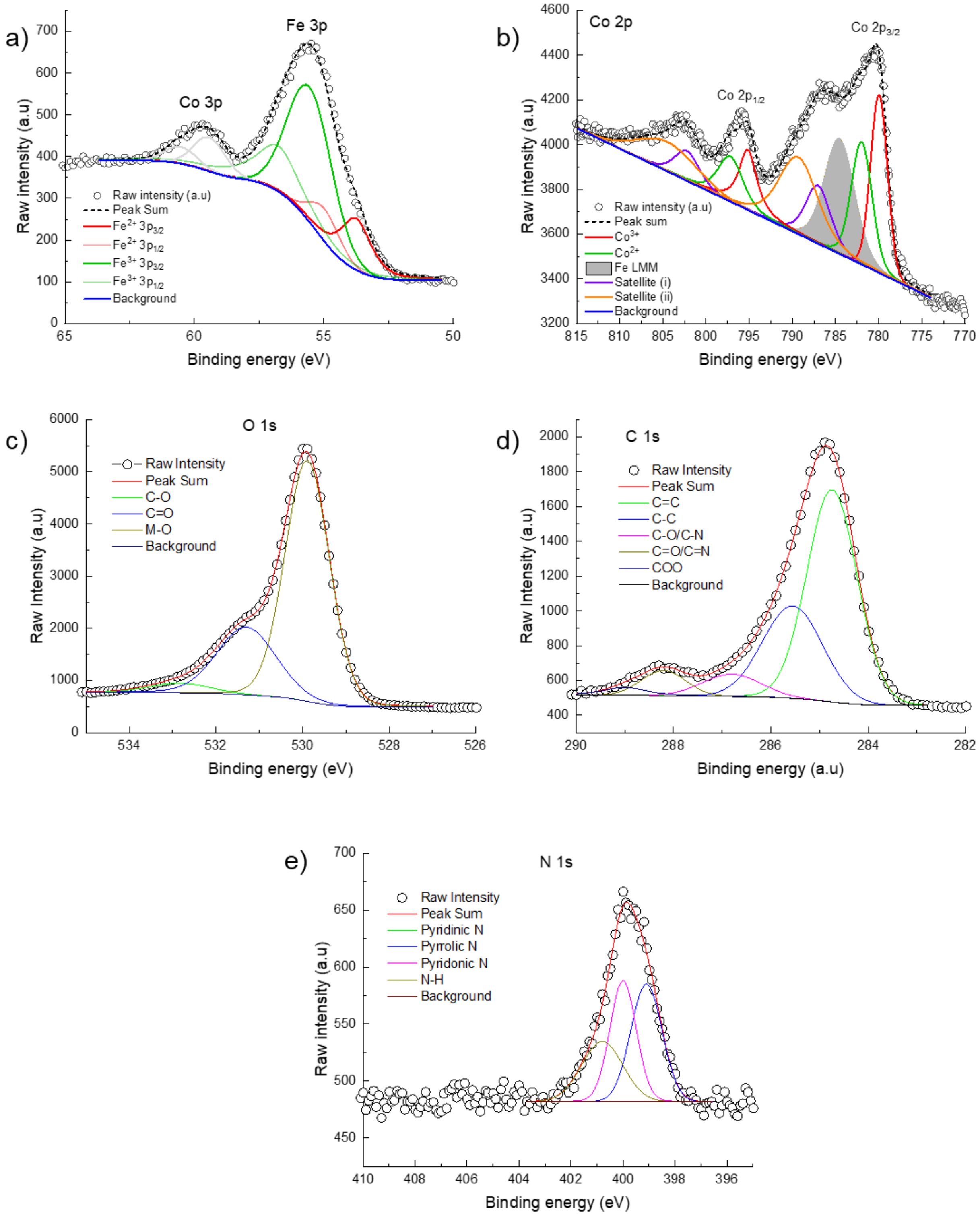

Figure S3. XPS analysis data for sample MNP@C (a) Fe 3p and Co 3p, (b) Co 2p, (c) O 1s, (d) C 1s and (e) N 1s.

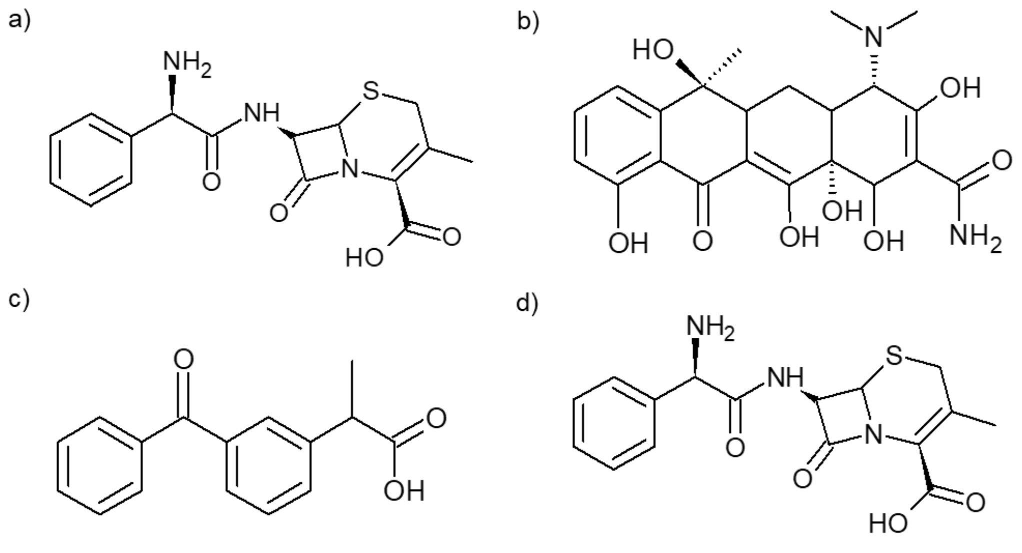

Figure S4. Molecular structure of amoxicillin (a), tetracycline (b), ketoprofen (c), and cephalexin (d).

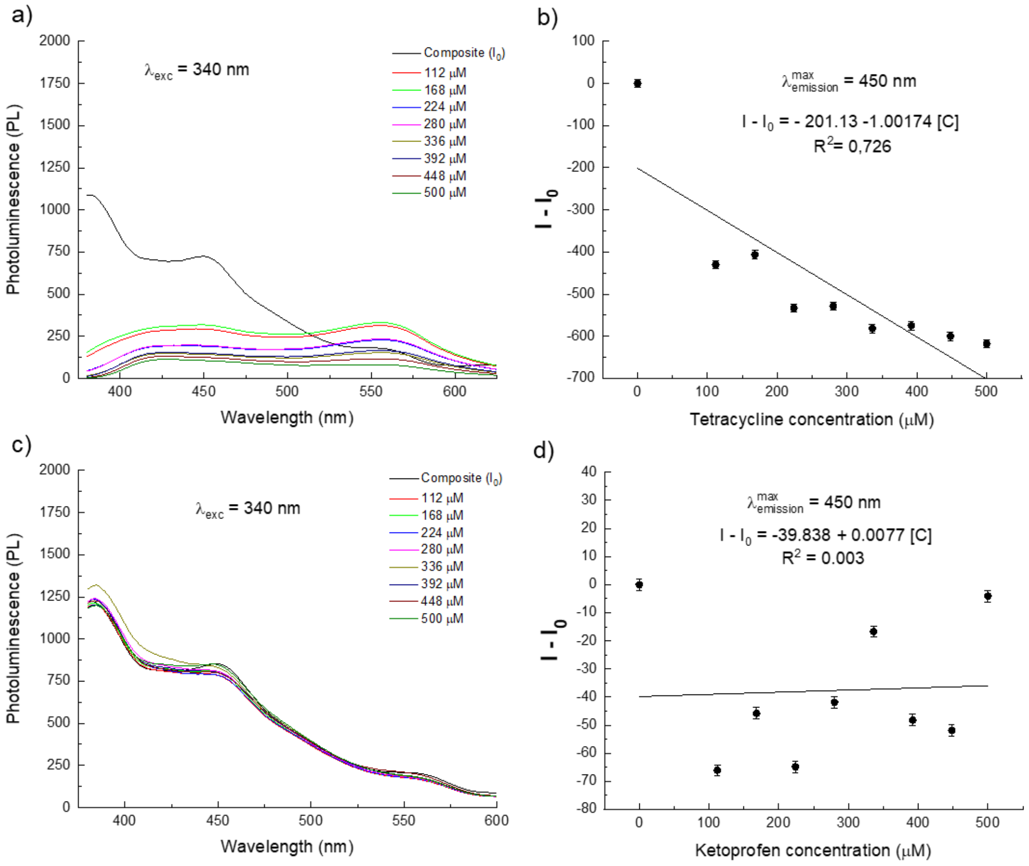

Figure S5. Emission spectra of MNP@C (λ_{exc}=340 nm) at different concentrations and plot of the I-I_0 as a function of the concentration, respectively, tetracycline (a-b), and ketoprofen (c-d).

Table S3. Studies on nanocomposites aimed at the detection of amoxicillin.

Material	Detection Method	Detection Range	LOD	Ref.
$FeCr_2O_4$ – MWCNTs	Voltammetry	0.1–70.0 μmol L^{-1}	0.05 μmol L^{-1}	[1]
SP-MIP	Chromatography	5–30 mg L^{-1}	0.147 mg L^{-1}	[2]
CuNPs and nanocomposite Cu-GO	Colorimetric	5 - 50 μmol/L	CuNPs: 2.17 μmol/L<br>Cu-GO: 1.71 μmol/L	[3]
	Spectrophotometry	0.01-1000 μmol/L	CuNPs: 0.0152 μmol/L<br>Cu-GO: 0.0012 μmol/L	
MIP-GO	Differential Pulse Voltammetry	5.0×10^{-10} - 9.1×10^{-10} mol/L	2.94×10^{-10} mol/L	[4]
PANI-AgBr	Cyclic voltammetry and chronoamperometry	0.193–0.855 nmol/L	0.193 nmol/L	[5]
$FeCr_2O_4$/TAPB-DMTP-COF	Differential pulse voltammetry	1 ~ 40 e 40 ~ 150 μmol/L	0.3 μmol/L	[6]

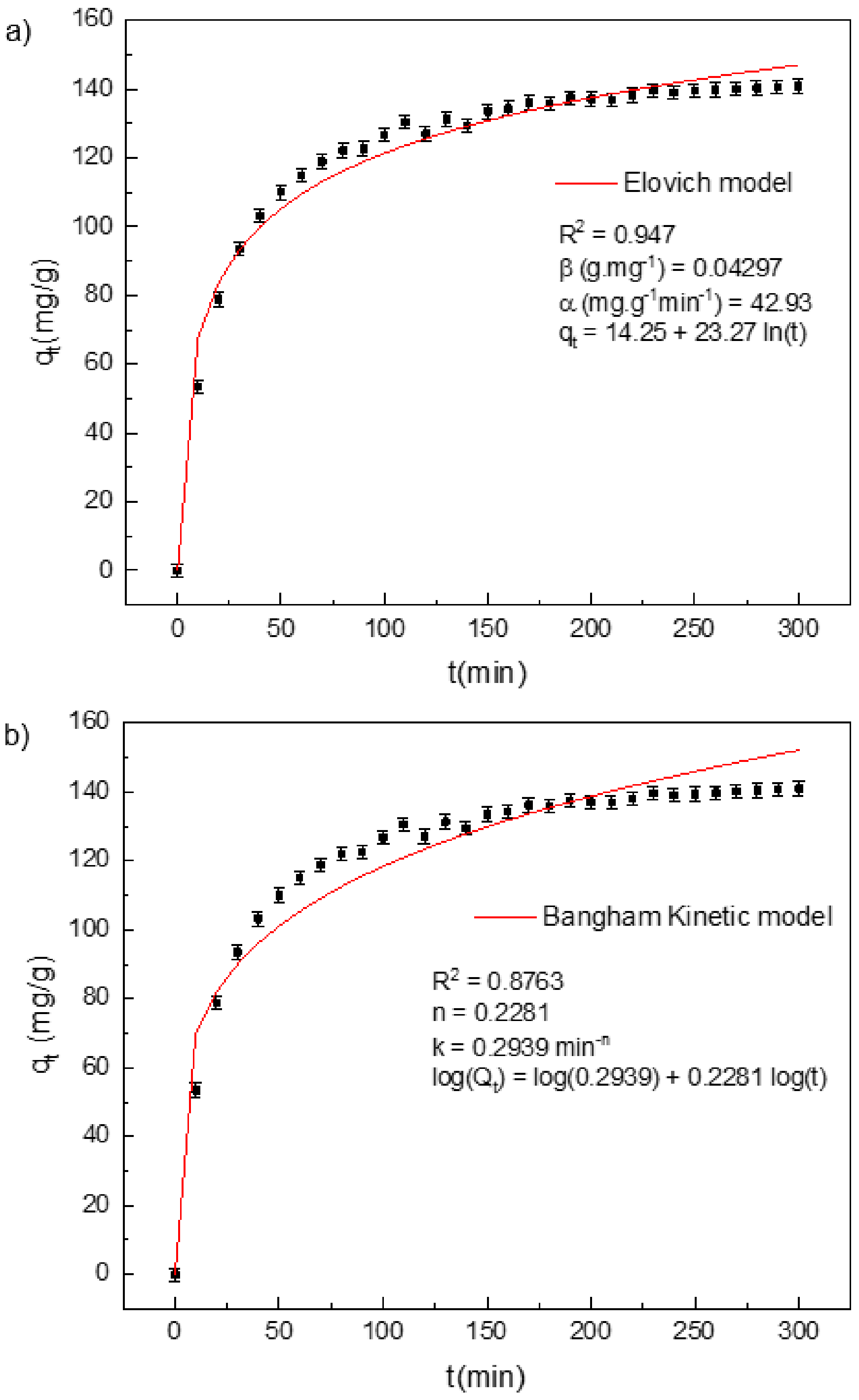

Figure S6. Fitting of adsorption kinetic data to the Elovich (a) and Bangham (b) model.

References

[1] A.A. Ensafi, A.R. Allafchian, B. Rezaei, Multiwall carbon nanotubes decorated with FeCr2O4, a new selective electrochemical sensor for amoxicillin determination, Journal of Nanoparticle Research 14 (2012) 1244. https://doi.org/10.1007/s11051-012-1244-3.

[2] R. López, S. Khan, S.E. Torres, A. Wong, M.D.P.T. Sotomayor, G. Picasso, Synthesis and Characterization of Magnetic Molecularly Imprinted Polymer for the Monitoring of Amoxicillin in Real Samples Using the Chromatographic Method, Magnetochemistry 9 (2023) 92. https://doi.org/10.3390/magnetochemistry9040092.

[3] N.T. Anh, N.X. Dinh, H. Van Tuan, M.Q. Doan, N.H. Anh, N.T. Khi, V.T. Trang, D.Q. Tri, A.-T. Le, Eco-friendly copper nanomaterials-based dual-mode optical nanosensors for ultrasensitive trace determination of amoxicillin antibiotics residue in tap water samples, Mater Res Bull 147 (2022) 111649. https://doi.org/10.1016/j.materresbull.2021.111649.

[4] S. Güney, T. Arslan, S. Yanık, O. Güney, An Electrochemical Sensing Platform Based on Graphene Oxide and Molecularly Imprinted Polymer Modified Electrode for Selective Detection of Amoxicillin, Electroanalysis 33 (2021) 46–56. https://doi.org/10.1002/elan.202060129.

[5] S. Palsaniya, T. Pal, S. Mukherji, Highly sensitive detection of amoxicillin by polyaniline-AgBr amperometry sensor: Fabrication and application in tap water and lake water, Chemical Engineering Journal 466 (2023) 143025. https://doi.org/10.1016/j.cej.2023.143025.

[6] Y. Yan, S. Zeng, F. Xu, J. Liao, H. Huang, Electrochemical sensor based on covalent organic framework loaded with FeCr2O4 nanoparticles for highly sensitive detection of amoxicillin, Journal of Solid State Electrochemistry 28 (2024) 2949–2957. https://doi.org/10.1007/s10008-024-05819-4.
